# Coded Caching Scheme for Multiaccess Cache-Assisted Partially Connected Linear Network via Multi-Antenna Placement Delivery Array

**DOI:** 10.3390/e28060580

**Published:** 2026-05-22

**Authors:** Yifei Huang, Siying Luo, Bowen Zheng

**Affiliations:** 1 The Key Lab of Education Blockchain and Intelligent Technology, Ministry of Education, Guangxi Normal University, Guilin 541004, China; hyf@guat.edu.cn (Y.H.); zhengbowen@stu.gxnu.edu.cn (B.Z.); 2 School of Science, Guilin University of Aerospace Technology, Guilin 541004, China; 3 Guangxi Academy of Artificial Intelligence, Guilin 541004, China

**Keywords:** coded caching, partially connected networks, MAPDA, normalized delivery time

## Abstract

In the traditional $\left(K,L,M_{T},M_{U},N\right)$ partially connected linear network, a central server stores a library of *N* files and connects to K+L−1 transmitters, each equipped with a cache of size $M_{T}$. Each user is connected to *L* neighboring transmitters and is equipped with a local cache of size $M_{U}$. Motivated by practical scenarios in which users can access multiple cache nodes, this paper considers a $\left(K,L,r,M_{T},M_{C},N\right)$ multiaccess cache-assisted partially connected linear network, where each user can access *r* neighboring cache nodes under a cyclic wrap-around topology, and each cache node has a storage capacity of $M_{C}$. We propose a general construction framework based on placement delivery arrays (PDAs). The analysis shows that, when the Maddah-Ali and Niesen (MN) scheme is employed and *r* is sufficiently large, the achieved normalized delivery time (NDT) approaches that of existing schemes for the traditional partially connected linear network. Moreover, under the same aggregate cache size accessible to each user, numerical results demonstrate that, as the cache size ratio increases, the gap between the NDT achieved by the proposed scheme and that of the traditional partially connected linear network scheme gradually diminishes, while the proposed scheme requires a smaller subpacketization level.

## 1. Introduction

The exponential growth of global data traffic, driven by the proliferation of smart devices and wireless infrastructure, imposes significant strain on wireless networks. Network traffic is typically characterized by pronounced peak and off-peak periods, leading to congestion during peak hours and resource underutilization otherwise. Coded caching [1] addresses this issue by prefetching content into local caches during off-peak periods and exploiting coding to generate multicast opportunities during peak periods, thereby reducing transmission load. This concept was first introduced by Maddah-Ali and Niesen (MN) in [1] for the shared-link network model. In this model, a central server with access to a library of *N* files is connected to *K* cache-aided users through an error-free shared link. Each user is equipped with a cache capable of storing up to *M* files. By transmitting coded multicast messages and leveraging cached content for interference cancellation, coded caching achieves not only the conventional local caching gain but also an additional coded multicasting gain. A coded caching system operates in two phases: the placement phase and the delivery phase. During the placement phase, which takes place in off-peak periods, the server populates the users’ caches without prior knowledge of future user demands. During the delivery phase, which occurs in peak periods, each user requests an arbitrary file from the library, and the server broadcasts coded packets such that each user can recover its requested file with the aid of its cached content.

Since the seminal work of Maddah-Ali and Niesen, coded caching has been extensively studied in various wireless network settings, including multiple-input single-output (MISO) broadcast channels [2,3,4,5,6,7,8], multiple-input multiple-output (MIMO) broadcast channels [9], single-input single-output (SISO) wireless channels [10,11,12,13,14,15,16,17], and MIMO wireless channels [18,19]. One of the major challenges in practical implementations is the reduction in the subpacketization level. To address this issue, refs. [4,5] proposed low-subpacketization schemes based on multi-antenna placement delivery arrays (MAPDAs).

For SISO interference channels, ref. [10] introduced a one-shot linear delivery scheme based on interference nulling. The work in [6] characterized the optimal normalized delivery time (NDT), originally introduced in [20], for certain cache size regimes. Reference [7] investigated a partially connected linear network in which each user is connected to only a subset of transmitters, and proposed a coded caching scheme that achieves the optimal NDT when the transmitter and user cache sizes are sufficiently large. However, the proposed scheme incurs high computational complexity. To alleviate this issue, ref. [8] developed coded caching schemes with reduced complexity based on MAPDAs.

However, the aforementioned models assume that each user can access only its dedicated local cache. In practical systems, the cache size available at end-user devices is often constrained by storage limitations. Furthermore, the schemes proposed in [7,8] require excessively high subpacketization levels to achieve low delivery latency (see Table 1 for the corresponding NDTs, subpacketization levels, and parameter constraints). Such high subpacketization levels may result in substantial packet management overhead, increased encoding and decoding complexity, and significant synchronization burden in practical implementations.

To overcome these limitations, a multiaccess caching model was introduced in [21], where users are allowed to retrieve contents from multiple neighboring cache nodes in a line network topology. By increasing the accessible aggregate cache size for each user, the multiaccess architecture provides additional flexibility for reducing delivery latency while potentially alleviating the subpacketization requirement.

In this paper, we investigate a $\left(K,L,r,M_{T},M_{C},N\right)$ multiaccess cache-assisted partially connected linear network with cyclic wrap-around connectivity among users and cache nodes, where the normalized delivery time (NDT) and subpacketization level are adopted as the performance metrics. There are *K* cache-less users, each of which can access *r* cache nodes in a cyclic wrap-around fashion. In this setting, the constructions proposed in [7,8] cannot be directly applied, because the cache contents accessible to adjacent users partially overlap, which violates the structural requirements of the delivery arrays employed in the existing schemes. Our main contributions are summarized as follows.

- We propose a construction framework for regular placement delivery arrays (PDAs). The proposed framework jointly constructs the node placement array, the user retrieval array, and the user delivery array. In particular, the proposed cyclic retrieval structure ensures that the contents retrieved by each user from its *r* accessible cache nodes are mutually non-overlapping, while preserving the MAPDA properties required to support multi-antenna transmission.
- Within this framework, the content retrieved by each user from its *r* associated cache nodes is guaranteed to be non-overlapping. For any given regular PDA, such as a $\left(t^{\prime }+1\right)$-$\left(K^{\prime },F^{\prime },Z^{\prime },S^{\prime }\right)$ PDA, the coded caching scheme obtained for the $\left(K=\left(r-1\right)K^{\prime }+t^{\prime },L,r,M_{T},M_{C},N\right)$ multiaccess cache-assisted partially connected linear network achieves $\frac{S^{\prime }\left(r-1\right)}{F^{\prime }L_{1}}$, where $L_{1}<r$.
- Compared with the traditional model based on the Maddah-Ali and Niesen (MN) scheme, the NDT ratio between our scheme and that in [8] (with $rt^{\prime }+L_{1}=K$) is $r/L_{1}$, where $L_{1}<r$. As $L_{1}\to r$, our performance approaches that of [8]. Compared with [7] and other schemes in [8], as the cache size ratio increases, our scheme approaches their performance with lower subpacketization.

**Notations:** Let bolded capital letters, bold lower case letter and curlicue font denote arrays, vectors and sets, respectively. We use |·| to denote the cardinality of a set. A set of consecutive integers is represented by [a:b]:={a,a+1,…,b}. For positive integers *a* and *b* with a≤b, ${\langle a\rangle }_{b}$ denotes the value of *a* modulo *b*; in particular, ${\langle a\rangle }_{b}=a\;\mathrm{mod}\;b$ for a<b, and ${\langle b\rangle }_{b}=b$. Let [a:b]t denote the collection of all *t*-sized subsets of [a:b]. Given an array P, let P(i,j) denote its entry of row *i* and column *j*. Let S[h] denote the *h*th smallest element of S, where h∈[1:|S|]. Let $\left[P^{\left(1\right)};P^{\left(2\right)};\ldots ;P^{\left(n\right)}\right]$ denote an array obtained by arranging arrays $P^{\left(1\right)};P^{\left(2\right)};\ldots ;P^{\left(n\right)}$ from top to bottom.

## 2. Partially Connected Network Placement Delivery Array

In this section, we first review the $\left(K,L,r,M_{T},M_{C},N\right)$ multiaccess cache-assisted partially connected linear network, along with existing schemes for the traditional $\left(K,L,M_{T},M_{U},N\right)$ model. We then review the definition of the multi-antenna placement delivery array (MAPDA).

### 2.1. System Model

**Figure 1:**
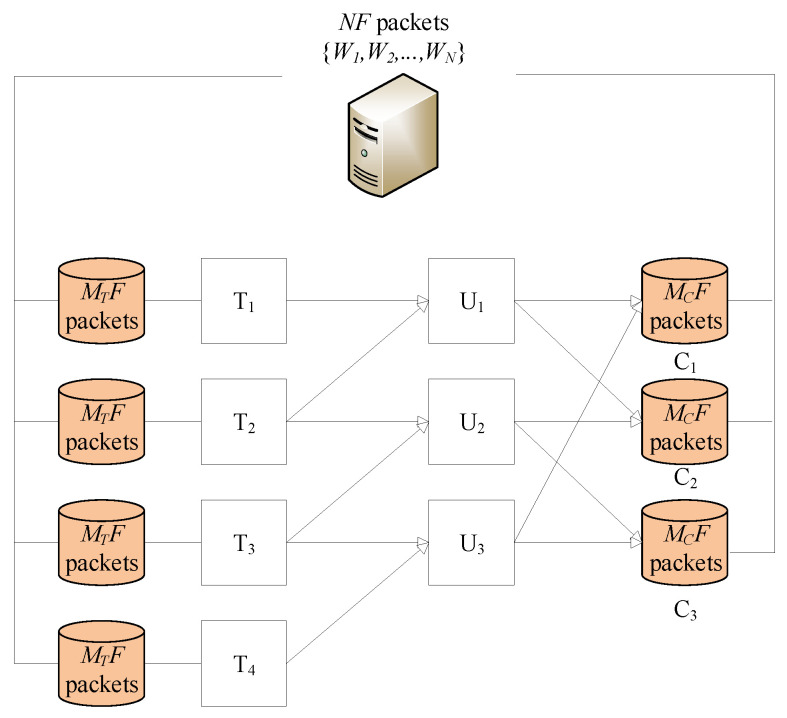
$\left(K=3,L=2,r=2,M_{T},M_{C},N\right)$ multiaccess cache-assisted partially connected linear network.

Consider a $\left(K,L,r,M_{T},M_{C},N\right)$ multiaccess cache-assisted partially connected linear network (see Figure 1). The system consists of a library of *N* files, denoted by $W=\{W_{1},\ldots ,W_{N}\}$, each of size *V* bits; K+L−1 linearly arranged transmitters, denoted by $T_{1},T_{2},\ldots ,T_{K+L-1}$; *K* linearly arranged users, denoted by $U_{1},U_{2},\ldots ,U_{K}$; and *K* cache nodes, denoted by $C_{1},C_{2},\ldots ,C_{K}$, each with a cache size of $M_{C}$ files, where $0\leq M_{C}\leq N/r$. Each user $U_{k}$, k∈[K], is connected to *L* consecutive transmitters $T_{k},T_{k+1},\ldots ,T_{k+L-1}$ and to *r* cache nodes $C_{k},C_{{\langle k+1\rangle }_{K}},\ldots ,C_{{\langle k+r-1\rangle }_{K}}$, where L≤K and r≤K. Here, *L* is referred to as the user connectivity. Figure 1 illustrates an example of such a multiaccess cache-assisted partially connected linear network with K=3, r=2, and L=2, where each transmitter is equipped with a finite-size cache and a single antenna. A $\left(K,L,r,M_{T},M_{C},N\right)$ multiaccess cache-assisted partially connected linear network (MA-CA-PCLN) coded caching scheme consists of two phases.

• **Placement Phase**: In this paper, we consider an uncoded placement strategy, where each node directly stores a subset of the library bits. Each file is divided into *F* packets; i.e., $W_{n}=\left(W_{n,1},W_{n,2},\ldots ,W_{n,F}\right)$, where each packet $W_{n,f}\in F_{2^{B}}$ for n∈[N] and f∈[F]. Here, *B* denotes the size (in bits) of each packet, and the file size is V=FB. Each transmitter and each cache node store a subset of packets from the library W, with cache capacities of at most $M_{T}F$ packets and $M_{C}F$ packets, respectively. Denote the cached contents at transmitter $T_{j}$ for j∈[K+L−1] and at cache node $C_{k}$ for k∈[K] as $Z_{T_{j}}$ and $Z_{C_{k}}$, respectively. User $U_{k}$ can access the cache contents of the *r* cache nodes to which it is connected, denoted as $Z_{U_{k}}={⋃}_{i=1}^{r}Z_{C_{{\langle k+i\rangle }_{K}}}$. We assume that the placement phase is performed without knowledge of future user demands.

• **Delivery Phase**: Each user $U_{k}$ requests an arbitrary file $W_{d_{k}}$ where $d_{k}\in \left[N\right]$ and k∈[K]. Let $d≜(d_{1},\ldots ,d_{K})$ denote the demand vector. According to the users’ demands and caches, each transmitter broadcasts the coded packets to its connected users. More precisely, each transmitter first uses a code for the Gaussian channel with the rate $B/\tilde{B}=\log P+o\left(\log P\right)$ to encode each packet into a coded packet as ${\tilde{W}}_{n,f}=\psi \left(W_{n,f}\right)\in C^{\tilde{B}}$, where ψ is the coding scheme for the Gaussian channel, e.g., random Gaussian coding. Here each coded packet contains $\tilde{B}$ complex symbols and carries one degree of freedom (DoF). The whole communication process contains *S* blocks, each of which consists of $\tilde{B}$ complex symbols (i.e., $\tilde{B}$ time slots). In each block s∈[S], the communication goal is to deliver a subset of the requested packets, denoted by $D_{s}=\left\{{\tilde{W}}_{d_{k_{1}},f_{1}},\ldots ,{\tilde{W}}_{d_{k_{|D_{s}|}}},f_{|D_{s}|}\right\}$, to a subset of users $K_{s}=\left\{k_{1},\ldots ,k_{|D_{s}|}\right\}$. Assume that the user $U_{k_{i}}$ requests the packet ${\tilde{W}}_{d_{k_{i}},f_{i}}$ for each $i\in [|D_{s}|]$. In this paper, we only consider linear coding schemes in the delivery phase. In each block s∈[S], each transmitter $T_{j}$, j∈[K+L−1], sends the linear combinations of the coded packets $x_{j}^{\left(s\right)}\in C^{\tilde{B}}$, i.e., $x_{j}^{\left(s\right)}=\sum_{i\in [|D_{s}|]}v_{j,k_{i}}^{\left(s\right)}{\tilde{W}}_{d_{k_{i}},f_{i}}$, where $v_{j,k_{i}}^{\left(s\right)}$ is the complex beam-forming coefficient and can be any complex value if the packet $W_{d_{k_{i}},f_{i}}$ is cached by transmitter $T_{j}$; otherwise $v_{j,k_{i}}^{\left(s\right)}=0$ for each $i\in [|D_{s}|]$. Then each user $U_{k}$, $k\in K_{s}$ receives the following signal

(1) $$y_{k}^{\left(s\right)}=\sum_{j=k}^{k+L-1}h_{k,j}^{\left(s\right)}x_{j}^{\left(s\right)}+\epsilon_{k}^{\left(s\right)},$$

through the wireless channel, where $h_{k,j}^{\left(s\right)}\in C$ denotes the channel coefficient from transmitter $T_{j}$ to user $U_{k}$, which is i.i.d. for different *k*, *j*, and *s*, and follows some continuous distribution (e.g., Rayleigh distribution). User $U_{k}\in K_{s}$ can decode the following coded signal ${\tilde{W}}_{d_{k},f}+\epsilon_{k}^{\left(s\right)}$ based on its can retrieve caches and received signal $y_{k}^{\left(s\right)}$. By assuming *P* is large enough, the coded packet ${\tilde{W}}_{d_{k},f}$ can be decoded with an error probability exponentially decreasing to zero.

To evaluate the transmission efficiency of the scheme, we adopt the same metric named normalized delivery time (NDT) as in [7,12], which is defined as

(2) $$\tau \left(M_{T},M_{U}\right)≜\lim_{P\to \infty }\lim_{V\to \infty }\sup\frac{\max_{d\in {\left[N\right]}^{K}}T}{V/\log P}$$

where *T* is the total time slots in the whole communication process. Since each file contains *F* packets, each of which has *B* bits, and there are a total of $S\tilde{B}$ time slots, (2) can be written as

(3) $$\tau =\lim_{P\to \infty }\lim_{V\to \infty }\frac{S\tilde{B}}{BF/\log P}=\lim_{P\to \infty }\frac{S}{F}\cdot \frac{\log P}{\log P+o(\log P)}=\frac{S}{F}.$$

### 2.2. Multi-Antenna Placement Delivery Array

This subsection briefly reviews the concept of the MAPDA and its relation to the coded caching scheme for the partially connected linear network.

**Definition** **1** ([4])**.** For positive integers L, K, F, Z and S, an F×K array P composed of a specific symbol “*” and integers in [S] is called an (L,K,F,Z,S) multiple-antenna placement delivery array (MAPDA) if it satisfies the following conditions:

*C1.*  *The symbol “∗” appears Z times in each column*.
*C2.*  *Each integer occurs at least once in the array*.
*C3.*  *Each integer s appears at most once in each column*.
*C4.*  *For any integer s∈[S], define $P^{\left(s\right)}$ to be the subarray of P including the rows and columns containing s, and let $r_{s}^{\prime }\times r_{s}$ denote the dimensions of $P^{\left(s\right)}$. The number of integer entries in each row of $P^{\left(s\right)}$ is less than or equal to L; i.e.,*
  (4) $$\begin{array}{c} \left|\{k_{1}\in \left[r_{s}\right]|\;P^{\left(s\right)}\left(f_{1},k_{1}\right)\in \left[S\right]\}\right|\leq L,\;\forall f_{1}\in \left[r_{s}^{\prime }\right]. \end{array}$$

If each integer appears *g* times in P, then P is regular, denoted by *g*-(L,K,F,Z,S) MAPDA. Let us take an example to further illustrate the concept of the MAPDA.

**Example** **1.** *We consider the following array:*

$$P=\begin{array}{c} \begin{array}{c} 1\;\;\;\;2\;\;\;\;3\;\;\;\;4 \end{array}\\ \left(\begin{array}{cccc} {}* & * & 1 & 1\\ 1 & 1 & * & * \end{array}\right) \end{array}.$$

*We can see that there are exactly S=1 integers, the star appears exactly once in each column, i.e., Z=1, and each integer occurs exactly g=4 times and occurs at most once in each column. Furthermore, in the array P, each row has exactly L=2 integer entries. By Definition 1, P is a 4-(2,4,2,2,2) MAPDA*.

Based on the MAPDA, the following result for coded caching schemes over partially connected linear networks was established in [8].

**Lemma** **1** ([8])**.** *Given an $\left(r,K,F_{1},Z_{1},S_{1}\right)$ MAPDA, there exists a $\left(K,L,M_{T},M_{U},N\right)$ coded caching scheme for the (K+L−1)×K partially connected linear network achieving the NDT $\tau =\frac{S_{1}}{F_{1}}$ with $\frac{LM_{T}}{N}=\frac{r}{\left\lceil K/L\right\rceil }\in Z^{+}$, $\frac{M_{U}}{N}=\frac{Z_{1}}{F_{1}}$, and subpacketization $F=LF_{1}$*.

Since our focus is on coded caching schemes over multiaccess networks, we adopt the node placement, user retrieve, and user delivery phases introduced in [22] to present the proposed scheme.

**Definition** **2.**

- *An F×Λ node placement array C consists of a star and null, where F and* Λ *represent the subpacketization and the number of cache nodes, respectively. For any integers j∈[F] and λ∈[Λ], the entry C(j,λ) is a star if and only if the cache node $C_{\lambda }$ caches the jth packet of each file*.
- *An F×K user retrieve array U consists of star and null, where F and K represent the subpacketization and the number of users respectively. For any integers j∈[F] and λ∈[Λ], the entry U(j,k) is a star if and only if the user k can retrieve the jth packet of each file from its connected cache nodes*.
- *An F×K user delivery array Q consists of {∗}∪[S], where the stars in Q have the same meaning as the stars in U. Each integer s∈[S] indexes the transmitted messages at block s. Integer S represents the total number of blocks in the delivery phase*.

By Lemma 1 and Definition 2, the construction of an MA-CA-PCLN scheme can be decomposed into the design of three components: node placement, user retrieval, and user delivery. In particular, we design the user delivery array Q as a multi-antenna placement delivery array (MAPDA).

## 3. Main Results

In this section, we present the main results of this work. We propose a PDA-based construction framework for the $\left(K,L,r,M_{T},M_{C},N\right)$ MA-CA-PCLN. Specifically, for any given PDA, the proposed framework constructs the corresponding node placement, user retrieval, and user delivery arrays, and yields the associated achievable normalized delivery time (NDT) and subpacketization level.

### 3.1. New Construction Framework

Since the connectivity between users and cache nodes follows a cyclic wrap-around topology, the resulting multiaccess structure is translation-invariant. This property enables us to exploit cyclic PDA structures to construct a structured placement scheme with uniform cache occupancy and analytically tractable performance.

For any given regular $\left(K^{\prime },F^{\prime },Z^{\prime },S^{\prime }\right)$ PDA P, *r* and *K* are such that $K=\left(r-1\right)K^{\prime }+t^{\prime }$, where $t^{\prime }=K^{\prime }Z^{\prime }/F^{\prime }$ denotes the number of ∗ entries per row of P. Define the set $F_{j}$ for each $j\in [F^{\prime }]$ as

(5) $$F_{j}=\left\{k^{\prime }\;|\;P\left(j,k^{\prime }\right)=*,\;k^{\prime }\in \left[1:K^{\prime }\right]\right\}.$$

That is, $F_{j}$ contains the column indices of all ∗ entries in the *j*-th row of P.

**Figure 2:**
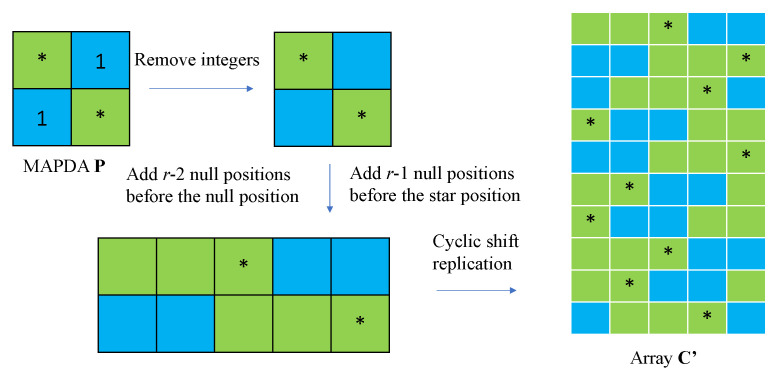
Example of the process of transforming the PDA into array $C^{\prime }$, with r=3.

Let $\beta =L_{1}/\gcd\left(r-1,L_{1}\right)$ where $L_{1}<r$. We then define the $(K\times F^{\prime }\times \beta )\times K$ array $C^{\prime }$ as

(6) $$\begin{array}{c} C^{\prime }\left(\left(j,m,k_{1}\right),k_{2}\right)=\left\{\begin{array}{cc} {}*, & \mathrm{if}\;k_{2}\in \left\{{\langle \left(r-1\right)F_{j}\left[i\right]+i+k_{1}-1\rangle }_{K}\;|\;i\in \left[1:t^{\prime }\right]\right\},\\ \mathrm{Null}, & \mathrm{otherwise}, \end{array}\right. \end{array}$$

where $j\in [F^{\prime }]$, m∈[β], $\beta =L_{1}/\gcd\left(r-1,L_{1}\right)$, $L_{1}<r$ and $k_{1},k_{2}\in \left[K\right]$. The construction idea of array $C^{\prime }$ is illustrated in Figure 2.

Since the connectivity between users and cache nodes follows a cyclic wrap-around network topology, the set of cache node indices accessible to user $U_{k}$, k∈[K], is given by $R_{k}=\left\{k,\;{\langle k+1\rangle }_{K},\;\ldots ,\;{\langle k+r-1\rangle }_{K}\right\}$. User $U_{k}$ can retrieve the cached contents from its connected cache nodes. Accordingly, the $(K\times F^{\prime }\times \beta )\times K$ array $U^{\prime }$ is defined as follows:

(7) $$U^{\prime }\left(\left(j,m,k_{1}\right),k_{2}\right)=\left\{\begin{array}{cc} {}*, & \mathrm{if}\;R_{k_{2}}\cap \left\{{\langle \left(r-1\right)F_{j}\left[i\right]+i+k_{1}-1\rangle }_{K}\;|\;i\in \left[1:t^{\prime }\right]\right\}\neq \emptyset ,\\ \mathrm{Null}, & \mathrm{otherwise}, \end{array}\right.$$

where $j\in [F^{\prime }]$, m∈[β] and $k_{1},k_{2}\in \left[K\right]$.

We adopt the same transmitter-side cache placement strategy as in [8]. Similarly to the cache-node placement, we use the matrix T to characterize the file storage at the transmitters. Let $\alpha =\gcd(K\times F^{\prime }\times \beta ,L)$; the $\left(K\times F^{\prime }\times \beta \times L/\alpha \right)\times \left(K+L-1\right)$ transmitter-placement array T is defined as

(8) $$T\left(\left(j,m,k_{1},l\right),k_{3}\right)=\left\{\begin{array}{cc} {}*, & \mathrm{if}\;{\langle k_{3}\rangle }_{L}\in \left\{{\langle l+\mu \rangle }_{L}\;|\;\mu \in \left[t\right]\right\},\\ \mathrm{Null}, & \mathrm{otherwise}, \end{array}\right.$$

where $t=LM_{T}/N\in \left[0:L\right]$, $j\in [F^{\prime }]$, m∈[β], $k_{1}\in \left[K\right]$, l∈[L/α] and $k_{3}\in \left[K+L-1\right]$.

Based on (6) and (7), we can determine that each row of the user retrieve array U contains exactly $\left(K^{\prime }-t^{\prime }\right)\left(r-1\right)$ null entries. Owing to the cyclic wrap-around network topology, these $\left(K^{\prime }-t^{\prime }\right)\left(r-1\right)$ null entries can be further partitioned into $\left(K^{\prime }-t^{\prime }\right)$ groups, each consisting of (r−1) entries. For each $\left(j,m\right)\in \left[F^{\prime }\right]\times \left[\beta \right]$ and each $\mu \in [1:K^{\prime }-t]$, we define a set of (r−1) column indices as

(9) $${\hat{U}}_{j,\mu }≜\left\{{\bar{F}}_{j}\left[\mu \right]r-\mu r+\left(\mu -1\right)\left(r-1\right)+q\;|\;q\in \left[1:r-1\right]\right\}\subseteq \left[1:K\right].$$

where ${\bar{F}}_{j}≜\left[1:K^{\prime }\right]\setminus F_{j}$ denotes the column index set of PDA **P** where the entries in row *j* are integers. Then define the set of row–column index pairs

(10) $$A_{j,\mu }≜\left\{\left((j,m),k\right)\;|\;m\in \left[\beta \right],\;k\in {\hat{U}}_{j,\mu }\right\}.$$

Partition $A_{j,\mu }$ into $G≜\frac{(r-1)\beta }{L_{1}}\in Z$ disjoint subsets ${\left\{A_{j,\mu ,\lambda }\right\}}_{\lambda =1}^{G}$ such that

(11) $$A_{j,\mu }=\overset{G}{\underset{\lambda =1}{⋃}}A_{j,\mu ,\lambda },\;\;A_{j,\mu ,\lambda }\cap A_{j,\mu ,\lambda^{\prime }}=\emptyset \;\left(\lambda \neq \lambda^{\prime }\right),\;\;\left|A_{j,\mu ,\lambda }\right|=L_{1}.$$

For each $k_{1}\in \left[K\right]$, define the $k_{1}$-shifted version of $A_{j,\mu ,\lambda }$ as

(12) $$A_{j,\mu ,\lambda }^{\left(k_{1}\right)}≜\left\{\left(\left(j,m,k_{1}\right),\;{\langle k+k_{1}-1\rangle }_{K}\right)\;|\;\left((j,m),k\right)\in A_{j,\mu ,\lambda }\right\},$$

Define $(K\times F^{\prime }\times \beta )\times K$ array $Q^{\prime }$ by

(13) $$Q^{\prime }\left(\left(j,m,k_{1}\right),k_{2}\right)=\left\{\begin{array}{cc} P\left(j,{\bar{F}}_{j}\left[\mu \right]\right)+\left(\lambda -1\right)S^{\prime }+\left(k_{1}-1\right)GS^{\prime },\; & \mathrm{if}\;\left(\left(j,m,k_{1}\right),k_{2}\right)\in A_{j,\mu ,\lambda }^{\left(k_{1}\right)},\\ {}*, & \mathrm{otherwise}, \end{array}\right.$$

for all $j\in [F^{\prime }]$, m∈[β], and $k_{1},k_{2}\in \left[K\right]$.

Using the above arrays $C^{\prime }$, $U^{\prime }$, and $Q^{\prime }$, the node placement array C and the user retrieve array U, Q can be constructed as $C=\underset{\underset{L}{︸}}{\left[C^{\prime };\ldots ;C^{\prime }\right]}$; $U=\underset{\underset{L}{︸}}{\left[U^{\prime };\ldots ;U^{\prime }\right]}$. We construct user delivery array Q by replicating $Q^{\prime }$$\frac{L}{\alpha }$ times vertically and then increasing the integers in $Q^{\prime }$ by the occurrence orders (from up to down) of $Q^{\prime }$; the $(K\times F^{\prime }\times \beta \times L/\alpha )\times K$ user delivery array Q is defined as

(14) $$Q=\left(\begin{array}{c} Q^{\prime }\\ Q^{\prime }+S\\ \vdots \\ Q^{\prime }+\left(\frac{L}{\alpha }-1\right)S \end{array}\right),$$

where $S=KS^{\prime }\left(r-1\right)\beta /L_{1}$.

As a result, each of C, U, and Q has $K\times F^{\prime }\times \beta \times L/\alpha$ rows. Together with the array T and Lemma 1, they yield the following result that characterizes the property of the proposed MA-CA-PCLN coded caching scheme.

**Theorem** **1.** *Given any $\left(t^{\prime }+1\right)-\left(K^{\prime },F^{\prime },Z^{\prime },S^{\prime }\right)$ PDA, there exists a $\left(K,L,r,M_{T},M_{C},N\right)$ multiaccess cache-assisted partially connected linear network scheme achieving the NDT $\tau =\frac{S^{\prime }\left(r-1\right)}{F^{\prime }L_{1}}$ with $\frac{LM_{T}}{N}=\frac{L_{1}}{\left\lceil K/L\right\rceil }\in Z^{+}$, $\frac{M_{C}}{N}=\frac{t^{\prime }}{K}$ and subpacketization $F=\frac{LKF^{\prime }\beta }{\alpha }$ with $K=\left(r-1\right)K^{\prime }+t^{\prime }$, $\beta =\frac{L_{1}}{\gcd(r-1,L_{1})}$, $\alpha =\gcd(KF^{\prime }\beta ,L)$ and $L_{1}<r$*.

**Proof.**  Since arrays C, U, and Q are obtained by copying $C^{\prime }$, $U^{\prime }$, and $Q^{\prime }$ respectively, we can obtain the structure of C, U, and Q by analyzing the structure of $C^{\prime }$, $U^{\prime }$, and $Q^{\prime }$.

Based on (6), we know that each row of $C^{\prime }$ contains $t^{\prime }$∗ symbols, each column contains $F^{\prime }t^{\prime }\beta$∗ symbols, and in each row, the distance between any two adjacent ∗s is at least r−1. Since each column in $C^{\prime }$ has a total of $KF^{\prime }\beta$ rows, the proportion of rows containing stars remains unchanged after copying to obtain C. Thus $M_{C}/N=F^{\prime }t^{\prime }\beta /\left(KF^{\prime }\beta \right)=t^{\prime }/K$. From (7), it follows that each row of $U^{\prime }$ contains $rt^{\prime }$∗ symbols. Hence, each row of $Q^{\prime }$ contains $rt^{\prime }$∗ symbols. Since the network topology follows a cyclic wrap-around structure, the construction employs the modulo-*K* cyclic shift mapping over the column indices, so that all columns have the same structure up to a cyclic shift. Therefore, each column of $Q^{\prime }$ contains $Z=rt^{\prime }\beta F^{\prime }$∗ symbols, which satisfies condition C1 of Definition 1.

From (9), we obtain (r−1) consecutive null positions in each row. Moreover, by (10), when replicating by a factor of β, these index positions are expanded to their corresponding replicated positions. According to the partition rule in (11), when $r-1\geq L_{1}$, there do not exist two entries ((j,m),k) and $\left(\left(j,m^{\prime }\right),k^{\prime }\right)$ in $A_{j,\mu ,\lambda }$ such that $m\neq m^{\prime }$ and $k=k^{\prime }$ simultaneously. By the filling rule in (13), any integer symbol *s* appears at most once in each column. Hence, conditions C2 and C3 of Definition 1 are satisfied. According to (13), the array $Q^{\prime }$ contains $KS^{\prime }\left(r-1\right)\beta /L_{1}$ distinct integers; i.e., $S=KS^{\prime }\left(r-1\right)\beta /L_{1}$. Based on (13), each integer occurs $L_{1}\left(t+1\right)$ times, since each integer occurs t+1 times and $|A_{j,\mu ,\lambda }|=L_{1}$.

Fix any integer *s*, and let $Q^{\prime (s)}$ be the subarray formed by the rows and columns of $Q^{\prime }$ that contain *s*, as in Definition 1. By construction, all occurrences of *s* are produced within the same block indexed by the same $k_{1}$ and λ in the row indices; hence, all rows in $Q^{\prime (s)}$ share the same $k_{1}$ and λ. Consider any row of $Q^{\prime (s)}$.

Assume that

$$Q\left((j,m,k_{1}),k\right)=Q\left(\left(j^{\prime },m^{\prime },k_{1}\right),k^{\prime }\right)=s.$$

We verify condition C4 by considering the following two steps.

**Step 1: $j=j^{\prime }$.** From (6) and (7), it follows that the parameter *m* only plays the role of replication and does not affect the positions of the ∗ entries. Hence, in Q, the rows indexed by $\left(j,m,k_{1}\right)$ and $\left(j^{\prime },m^{\prime },k_{1}\right)$ have exactly the same ∗ pattern. Moreover, by (13), since
  $$|A_{j,\mu ,\lambda }^{\left(k_{1}\right)}|=L_{1},$$
  for a fixed *j*, the integer *s* appears exactly $L_{1}$ times, namely, in exactly $L_{1}$ columns. Therefore, in the induced subarray $Q^{\prime (s)}$, each of the rows $\left(j,m,k_{1}\right)$ and $\left(j^{\prime },m^{\prime },k_{1}\right)$ contains exactly $L_{1}$ non-star entries caused by the integer *s*.
**Step 2: $j\neq j^{\prime }$.** In this case, it suffices to prove that
  $$Q\left(\left(j,m,k_{1}\right),k^{\prime }\right)=Q\left((j^{\prime },m^{\prime },k_{1}),k\right)=*.$$
  Indeed, by Case 1, we have already shown that for a fixed *j*, each row contains exactly $L_{1}$ integer positions caused by the symbol *s*. Therefore, if no additional integer entries are introduced into the row $\left(j,m,k_{1}\right)$ from any other row index $j^{\prime }\neq j$, then every row of $Q^{\prime (s)}$ contains exactly $L_{1}$ integer entries, which is precisely the requirement of condition C4. Now, since
  $$P\left(j,{\bar{F}}_{j}\left[\mu \right]\right)=P\left(j^{\prime },{\bar{F}}_{j^{\prime }}\left[\mu^{\prime }\right]\right)=s$$
  P is a PDA. The PDA property directly implies that the corresponding cross positions must be star entries; i.e.,
  $$P\left(j,{\bar{F}}_{j^{\prime }}\left[\mu^{\prime }\right]\right)=P\left(j^{\prime },{\bar{F}}_{j}\left[\mu \right]\right)=*.$$
  Equivalently, we have
  $${\bar{F}}_{j}\left[\mu \right]\in F_{j^{\prime }}\;\mathrm{and}\;{\bar{F}}_{j^{\prime }}\left[\mu^{\prime }\right]\in F_{j}.$$
  By (7), for any fixed row $\left(j,m,k_{1}\right)$, the null-column indices in U are generated by the elements in ${\bar{F}}_{j}$ through the mapping ${\hat{U}}_{j,\mu }$. In other words, ${\hat{U}}_{j,\mu }$ is derived from $\bar{F}j$ such that for any $k\in \hat{U}j,\mu$, we have $U(\left(j,m,k\right),k_{1})=\mathrm{Null}$; otherwise, $U(\left(j,m,k\right),k_{1})=*$. Since ${\bar{F}}_{j^{\prime }}\left[\mu^{\prime }\right]\in F_{j}$, the column index $k^{\prime }\notin {\hat{U}}_{j,\mu }$. Therefore, this entry remains ∗ in Q; that is,
  $$Q\left(\left(j,m,k_{1}\right),k^{\prime }\right)=*.$$
  Similarly, because ${\bar{F}}_{j}\left[\mu \right]\in F_{j^{\prime }}$, we also have
  $$Q\left((j^{\prime },m^{\prime },k_{1}),k\right)=*.$$
  Consequently, every row of the induced subarray $Q^{\prime (s)}$ contains exactly $L_{1}$ non-ast entries. Hence, condition C4 is satisfied. Combining the above arguments, $Q^{\prime }$ satisfies C1–C4 and is thus an
  $$\left(L_{1},\;K=K^{\prime }\left(r-1\right)+t^{\prime },\;F=K\beta F^{\prime },\;Z=rt\beta F^{\prime },\;S=KS^{\prime }\left(r-1\right)\beta /L_{1}\right)\;\mathrm{MAPDA}.$$
  Hence, Q is an
  $$\left(L_{1},\;K=K^{\prime }\left(r-1\right)+t^{\prime },\;F=\frac{LK\beta F^{\prime }}{\alpha },\;Z=\frac{Lrt\beta F^{\prime }}{\alpha },\;S=\frac{LKS^{\prime }\left(r-1\right)\beta }{L_{1}\alpha }\right)\;\mathrm{MAPDA}.$$
  Consequently, applying Lemma 1 completes the proof.    □

### 3.2. Performance Evaluation

In this subsection, we compare the performance of the proposed construction, instantiated from the MN PDA scheme, with the schemes in Table 1. Based on Theorem 1 and the MN PDA construction, we obtain the following scheme.

**Corollary** **1.** *Given any (t+1)−(K′,K′t,K′−1t−1,K′t+1) PDA, there exists a $\left(K,L,r,M_{T},M_{C},N\right)$ multiaccess cache-assisted partially connected linear network scheme achieving the NDT $\tau =\frac{\left(K^{\prime }-t\right)\left(r-1\right)}{\left(t+1\right)L_{1}}$ with $\frac{M_{T}}{N}=\frac{L_{1}}{L\lceil K(r-1)+t/L\rceil }$, $\frac{M_{C}}{N}=\frac{t}{K(r-1)+t}$ and subpacketization F=LK′K′tβα with $K=\left(r-1\right)K^{\prime }+t$, $\beta =\frac{L_{1}}{\gcd(r-1,L_{1})}$, α=gcd(KKtβ,L) and $L_{1}<r$*.

We take the scheme in Corollary 1 to perform a theoretical comparison of the proposed scheme with CXHZW scheme 1 in Table 1. Since CXHZW scheme 1 is developed for the traditional model, while each user in our setting can access *r* cache nodes, the total cache size available to each user is $rM_{C}$. Hence, for a fair comparison, the user–cache ratio in the traditional model is set to $\frac{M_{U}}{N}=\frac{rM_{C}}{N}$, i.e., $\frac{z}{K^{\prime }}=\frac{rt}{K(r-1)+t}$. Accordingly, the ratio of the NDT achieved by Corollary 1 to that of CXHZW scheme 1 is given by

(15) $$\begin{array}{cc} \frac{\tau_{Cor.1}}{\tau_{Scheme1}} & =\frac{\left(K-t)(r-1\right)}{L_{1}\left(t+1\right)}/\frac{K^{\prime }-z}{K^{\prime }}\\ \; & =\frac{K(r-1)+t}{L_{1}\left(t+1\right)}\\ \; & \leq \frac{1+t\frac{r}{r-1}}{t+1}\frac{r-1}{L_{1}}\;\;\;\;\;\;\left(K^{\prime }=z+L_{1}\right)\\ \; & \leq \frac{r}{L_{1}}\;\;\;\;\;\;\left(L_{1}<r\right) \end{array}$$

According to (15) it can be seen that the NDT of our scheme does not exceed $r/L_{1}$ times that of the scheme in [8] due to the constraints imposed by the topological network: the ratio is generally within *r* times. In the case where $L_{1}=r-1$, the performance of our scheme approaches that of the scheme in [8] when *r* is sufficiently large.

Furthermore, we conduct numerical comparisons between the scheme in Corollary 1 and CXHZW scheme 2 as well as the XTZ scheme.

**Figure 3:**
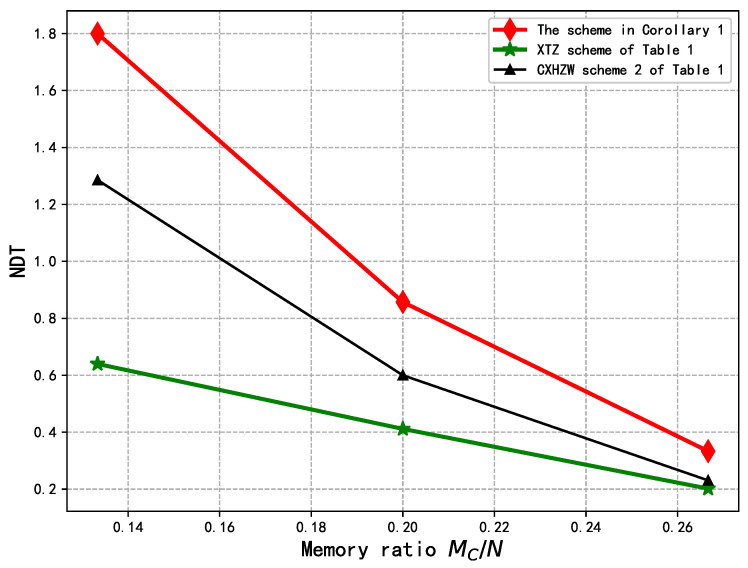
Achievable NDTs of the XTZ scheme and the CXHZW scheme 2 in Table 1, and the scheme in Corollary 1 when K=30, L=18, $L_{1}=2$, r=3 and $M_{T}/N=0.05$.

**Figure 4:**
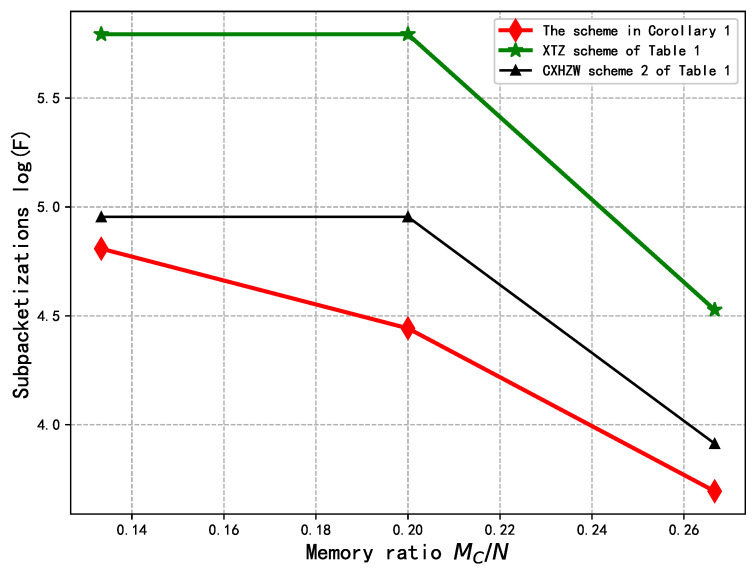
Achievable subpacketizations of the XTZ scheme and the CXHZW scheme 2 in Table 1, and the scheme in Corollary 1 when K=30, L=18, $L_{1}=2$, r=3 and $M_{T}/N=0.05$.

From Figure 3, it can be observed that the NDT achieved by the scheme in Corollary 1 is strictly within a factor of r=3 of the XTZ scheme and CXHZW scheme 2. Moreover, as the cache size ratio at the cache nodes increases, the achieved NDT approaches that of the existing schemes. However, from Figure 4, the scheme in Corollary 1 achieves a smaller subpacketization level than the XTZ scheme and CXHZW scheme 2.

### 3.3. Example of Theorem 1

Consider a $\left(K^{\prime },F^{\prime },Z^{\prime },S^{\prime }\right)=\left(2,2,1,1\right)$ PDA P as follows:

$$\begin{array}{c} P=\left(\begin{array}{cc} {}* & 1\\ 1 & * \end{array}\right). \end{array}$$

Let L=5, r=3 and $L_{1}=2$. Then we obtain $K=K^{\prime }\left(r-1\right)+t^{\prime }=5$ and $\frac{L_{1}}{\left\lceil K/L\right\rceil }=\frac{2}{\left\lceil 5/5\right\rceil }=2\in Z^{+}$. Accordingly, we have $M_{T}/N=2/L=2/5$ and $M_{C}/N=t^{\prime }/K=1/5$. Therefore, we can construct a $\left(K,L,r,M_{T},M_{C},N\right)=\left(5,5,3,4,2,10\right)$ MA-CA-PCLN coded caching scheme.

• **Placement Phase**: We can obtain $\beta =L_{1}/\gcd\left(r-1,L_{1}\right)=1$ and $\alpha =\gcd(5\times 2,5=\gcd\left(KF^{\prime }\beta ,L\right)=5$. Since $K^{\prime }\left(r-1\right)+t=5$, each file is divided into $K\times F^{\prime }\times L/\alpha =5\times 2\times 5/5=10$ packets; i.e., $W_{n}={\left(W_{n,j,m,l}^{k_{1}}\right)}_{j\in \left[2\right],m\in \left[1\right],l\in \left[1\right],k_{1}\in \left[5\right]}$ where n∈[N]. For brevity, the indices *m* and *l* are omitted in the subsequent representation; i.e., $W_{n}={\left(W_{n,j}^{k_{1}}\right)}_{j\in \left[2\right],k_{1}\in \left[5\right]}$. From (6) we can obtain the following 10×5 array $C^{\prime }$:

$$\begin{array}{c} C^{\prime }=\left(\begin{array}{ccccc} \;□ & □ & * & □ & □\\ \;□ & □ & □ & □ & *\\ \;□ & □ & □ & * & □\\ \;* & □ & □ & □ & □\\ \;□ & □ & □ & □ & *\\ \;□ & * & □ & □ & □\\ \;* & □ & □ & □ & □\\ \;□ & □ & * & □ & □\\ \;□ & * & □ & □ & □\\ \;□ & □ & □ & * & □ \end{array}\right)\begin{array}{c} \left(1,1\right)\\ \left(2,1\right)\\ \left(1,2\right)\\ \left(2,2\right)\\ \left(1,3\right)\\ \left(2,3\right)\\ \left(1,4\right)\\ \left(2,4\right)\\ \left(1,5\right)\\ \left(2,5\right) \end{array}. \end{array}$$

Since L/α=1, the node-placement array $C=C^{\prime }$. From the node-placement array C, each cache node $Z_{C_{k}}$ where k∈[K] caches the following packets:

$$\begin{array}{c} Z_{C_{k}}=\left\{W_{n,j}^{k_{1}}|C\left(\left(j,m,k_{1},l\right),k\right)=*,j\in \left[F^{\prime }\right],m\in \left[\beta \right],l\in \left[L/\alpha \right],k_{1}\in \left[K\right],n\in \left[N\right]\right\}. \end{array}$$

Thus cache nodes store the following packets:

$$\begin{array}{cc} Z_{C_{1}} & =\left\{W_{n,1}^{4},W_{n,2}^{2}|n\in \left[10\right]\right\},Z_{C_{2}}=\left\{W_{n,2}^{3},W_{n,1}^{5}|n\in \left[10\right]\right\},\\ Z_{C_{3}} & =\left\{W_{n,1}^{1},W_{n,2}^{4}|n\in \left[10\right]\right\},Z_{C_{4}}=\left\{W_{n,1}^{2},W_{n,2}^{5}|n\in \left[10\right]\right\},\\ Z_{C_{5}} & =\{W_{n,1}^{3},W_{n,2}^{1}|n\in \left[10\right]\}. \end{array}$$

Each user can retrieve cached contents from r=3 consecutive cache nodes. For example, user $U_{1}$ can access cache nodes $C_{1}$, $C_{2}$ and $C_{3}$. The retrievable contents of the user are given as follows:

$$\begin{array}{c} Z_{U_{1}}=\left\{W_{n,1}^{1},W_{n,2}^{2},W_{n,2}^{3},W_{n,1}^{4},W_{n,2}^{4},W_{n,2}^{5},|n\in \left[10\right]\right\},\\ Z_{U_{2}}=\left\{W_{n,1}^{1},W_{n,1}^{2},W_{n,2}^{3},W_{n,2}^{4},W_{n,1}^{5},W_{n,2}^{5},|n\in \left[10\right]\right\},\\ Z_{U_{3}}=\left\{W_{n,1}^{1},W_{n,2}^{1},W_{n,1}^{2},W_{n,1}^{3},W_{n,2}^{4},W_{n,2}^{5},|n\in \left[10\right]\right\},\\ Z_{U_{4}}=\left\{W_{n,2}^{1},W_{n,1}^{2},W_{n,2}^{2},W_{n,1}^{3},W_{n,1}^{4},W_{n,2}^{5},|n\in \left[10\right]\right\},\\ Z_{U_{5}}=\left\{W_{n,2}^{1},W_{n,2}^{2},W_{n,1}^{3},W_{n,2}^{3},W_{n,1}^{4},W_{n,1}^{5},|n\in \left[10\right]\right\}. \end{array}$$

From (7) we can obtain the following 10×5 array $U^{\prime }$:

(16) $$\begin{array}{c} U^{\prime }=\left(\begin{array}{ccccc} \;* & * & * & □ & □\\ \;□ & □ & * & * & *\\ \;□ & * & * & * & □\\ \;* & □ & □ & * & *\\ \;□ & □ & * & * & *\\ \;* & * & □ & □ & *\\ \;* & □ & □ & * & *\\ \;* & * & * & □ & □\\ \;* & * & □ & □ & *\\ \;□ & * & * & * & □ \end{array}\right)\begin{array}{c} \left(1,1\right)\\ \left(2,1\right)\\ \left(1,2\right)\\ \left(2,2\right)\\ \left(1,3\right)\\ \left(2,3\right)\\ \left(1,4\right)\\ \left(2,4\right)\\ \left(1,5\right)\\ \left(2,5\right) \end{array}. \end{array}$$

From (16), we observe that the array contains $3=rt^{\prime }=3\times 1$∗ entries in each row and $6=rt^{\prime }\times F^{\prime }=3\times 2$∗ entries in each column, which is consistent with our preceding results.

Since $t=LM_{T}/N=2$, K+L−1=9, from (8) we can obtain the following 10×9 transmitter-placement array T:

$$\begin{array}{c} T=\left(\begin{array}{ccccccccc} \;* & * & □ & □ & □ & * & * & □ & □\\ \;* & * & □ & □ & □ & * & * & □ & □\\ \;□ & * & * & □ & □ & □ & * & * & □\\ \;□ & * & * & □ & □ & □ & * & * & □\\ \;□ & □ & * & * & □ & □ & □ & * & *\\ \;□ & □ & * & * & □ & □ & □ & * & *\\ \;□ & □ & □ & * & * & □ & □ & □ & *\\ \;□ & □ & □ & * & * & □ & □ & □ & *\\ \;* & □ & □ & □ & * & * & □ & □ & □\\ \;* & □ & □ & □ & * & * & □ & □ & □ \end{array}\right)\begin{array}{c} \left(1,1\right)\\ \left(2,1\right)\\ \left(1,2\right)\\ \left(2,2\right)\\ \left(1,3\right)\\ \left(2,3\right)\\ \left(1,4\right)\\ \left(2,4\right)\\ \left(1,5\right)\\ \left(2,5\right) \end{array}, \end{array}$$

From transmitter-placement array T, each cache node $Z_{T_{k}}$ where k∈[K+L1] caches the following packets:

$$\begin{array}{c} Z_{T_{k}}=\left\{W_{n,j}^{k_{1}}|T\left(\left(j,m,k_{1},l\right),k\right)=*,j\in \left[F^{\prime }\right],m\in \left[\beta \right],l\in \left[L/\alpha \right],k_{1}\in \left[K\right],n\in \left[N\right]\right\}. \end{array}$$

Thus the transmitters cache respectively store the following packets:

$$\begin{array}{cc} Z_{T_{1}} & =\left\{W_{n,1}^{1},W_{n,2}^{1},W_{n,1}^{5},W_{n,2}^{5}|n\in \left[10\right]\right\},Z_{T_{2}}=\left\{W_{n,1}^{1},W_{n,2}^{1},W_{n,1}^{2},W_{n,2}^{2}|n\in \left[10\right]\right\},\\ Z_{T_{3}} & =\left\{W_{n,1}^{2},W_{n,2}^{2},W_{n,1}^{3},W_{n,2}^{3}|n\in \left[10\right]\right\},Z_{T_{4}}=\left\{W_{n,1}^{3},W_{n,2}^{3},W_{n,1}^{4},W_{n,2}^{4}|n\in \left[10\right]\right\},\\ Z_{T_{5}} & =\left\{W_{n,1}^{4},W_{n,2}^{4},W_{n,1}^{5},W_{n,2}^{5}|n\in \left[10\right]\right\},Z_{T_{6}}=\left\{W_{n,1}^{1},W_{n,2}^{1},W_{n,1}^{5},W_{n,2}^{5}|n\in \left[10\right]\right\},\\ Z_{T_{7}} & =\left\{W_{n,1}^{1},W_{n,2}^{1},W_{n,1}^{2},W_{n,2}^{2}|n\in \left[10\right]\right\},Z_{T_{8}}=\left\{W_{n,1}^{2},W_{n,2}^{2},W_{n,1}^{3},W_{n,2}^{3}|n\in \left[10\right]\right\},\\ Z_{T_{9}} & =\{W_{n,1}^{3},W_{n,2}^{3},W_{n,1}^{4},W_{n,2}^{4}|n\in \left[10\right]\}. \end{array}$$

• **Delivery Phase**: Assume that the demand vector is d≜(1,…,K); i.e., user $U_{k}$ requests file $W_{k}$ for k∈[5]. From (13) we can obtain the following 10×5 array $Q^{\prime }$:

$$\begin{array}{c} Q^{\prime }=\left(\begin{array}{ccccc} \;* & * & * & 1 & 1\\ \;1 & 1 & * & * & *\\ \;2 & * & * & * & 2\\ \;* & 2 & 2 & * & *\\ \;3 & 3 & * & * & *\\ \;* & * & 3 & 3 & *\\ \;* & 4 & 4 & * & *\\ \;* & * & * & 4 & 4\\ \;* & * & 5 & 5 & *\\ \;5 & * & * & * & 5 \end{array}\right)\begin{array}{c} \left(1,1\right)\\ \left(2,1\right)\\ \left(1,2\right)\\ \left(2,2\right)\\ \left(1,3\right)\\ \left(2,3\right)\\ \left(1,4\right)\\ \left(2,4\right)\\ \left(1,5\right)\\ \left(2,5\right) \end{array}, \end{array}$$

Since the user delivery array $Q=Q^{\prime }$, Q is an $\left(L_{1}=2,K=5,F=10,Z=6,S=5\right)$ MAPDA. Based on Q, the entire communication process is completed in S=5 time slots. Consider time slot 1. User $U_{1}$ receive the coded signals

$$x_{1}^{\left(1\right)}=W_{1,2}^{1}$$

enabling to decode the packets $W_{1,2}^{1}$. Users $U_{2}$ and $U_{4}$ receive the coded signal

$$x_{6}^{\left(1\right)}=W_{2,2}^{1}⊕W_{4,1}^{1}.$$

$U_{2}$ and $U_{4}$ can retrieve packets $W_{4,1}^{1}$ and $W_{2,2}^{1}$ respectively, thus enabling them to decode the packets $W_{2,2}^{1}$ and $W_{4,1}^{1}$. User $U_{5}$ receives the coded signals

$$x_{7}^{\left(1\right)}=W_{5,1}^{1}$$

enabling it to decode the packets $W_{5,1}^{1}$. The overall delivery strategy is shown in Table 2. From (2) and (3), we can obtain the NDT $\tau =\frac{S}{F}=\frac{5}{10}=\frac{1}{2}<\tau_{XTZ}=0.4$, but F/FXTZ=10/53(5−3)=1/2.

## 4. Conclusions

In this paper, we study a $\left(K,L,r,M_{T},M_{C},N\right)$ multiaccess cache-assisted partially connected linear network with a cyclic wrap-around connectivity pattern between users and cache nodes. We propose a construction framework under which the placement strategy at the cache nodes in our scheme achieves maximum utilization of the cache capacity. Under the same aggregate cache size accessible to each user, numerical results show that as the cache size ratio increases, the gap between the NDT achieved by the proposed scheme and that of existing schemes for the traditional partially connected linear network diminishes, while the proposed scheme achieves a smaller subpacketization.

## Figures and Tables

**Table 1:** The schemes with NDT and subpacketization in [7,8] with $K,t\in N^{+}$, $\beta =sgn\left(\frac{t}{m}+1,\frac{m}{r}\right)+\frac{r-m}{m}$ and sgn(x,y) equal to 1 if y=1, and x otherwise.

Scheme	NDT	Subpacketization	Parameter Limitations
	$\tau =\left(1-\frac{1}{L}+\frac{N}{M_{U}L+N}\right)\left(1-\frac{M_{U}}{N}\right)$	F=LMULNL	$\frac{M_{T}L}{N}=1,0\leq \frac{M_{U}L}{N}\leq L-1$
XTZ Scheme [7]	$\tau =\frac{1-\frac{M_{U}}{N}}{\min\left\{\frac{M_{T}}{N}+\frac{M_{U}}{N},\;1\right\}}$	LMULNL−MULN	$\frac{LM_{T}}{N}\in \left[2:L\right],\frac{M_{T}}{N}+\frac{M_{U}}{N}\geq 1$
	$\tau =\frac{1-\frac{M_{U}}{N}}{\min\left\{\frac{M_{T}}{N}+\frac{M_{U}}{N},\;1\right\}}$	LMULNL−MULN−1MTLN−1LN−MULN	$\;\;\;\;\;\;\;\;\frac{M_{T}}{N}+\frac{M_{U}}{N}<1$
CXHZW Scheme 1 [8]	$\tau =\frac{K-t}{t+r}$	F=LK	t+r=K
CXHZW Scheme 2 [8]	$\tau =\frac{K-t}{t-m}\frac{sgn(\frac{t}{m}+1,\frac{m}{r})}{\beta }$	LβK/mt/m	t+r<K,m≤r

**Table 2:** Delivery strategy.

Time Slot	Coded Signal	Transmitter
1	$x_{1}^{\left(1\right)}=W_{1,2}^{1}$	$T_{1}$
1	$x_{6}^{\left(1\right)}=W_{2,2}^{1}⊕W_{4,1}^{1}$	$T_{6}$
1	$x_{7}^{\left(1\right)}=W_{5,1}^{1}$	$T_{7}$
2	$x_{2}^{\left(2\right)}=W_{1,1}^{2}⊕W_{2,2}^{2}$	$T_{2}$
2	$x_{3}^{\left(2\right)}=W_{3,2}^{2}$	$T_{3}$
2	$x_{7}^{\left(2\right)}=W_{5,1}^{2}$	$T_{7}$
3	$x_{3}^{\left(3\right)}=W_{1,1}^{3}⊕W_{3,2}^{3}$	$T_{3}$
3	$x_{4}^{\left(3\right)}=W_{3,1}^{3}⊕W_{4,2}^{3}$	$T_{4}$
4	$x_{4}^{\left(4\right)}=W_{2,1}^{4}⊕W_{4,2}^{4}$	$T_{4}$
4	$x_{5}^{\left(4\right)}=W_{3,1}^{4}⊕W_{5,2}^{4}$	$T_{5}$
5	$x_{1}^{\left(5\right)}=W_{1,2}^{5}$	$T_{1}$
5	$x_{5}^{\left(5\right)}=W_{3,1}^{5}⊕W_{5,2}^{5}$	$T_{5}$
5	$x_{6}^{\left(5\right)}=W_{4,1}^{5}$	$T_{6}$

## Data Availability

The data are available upon request from the corresponding author.
